# High-Level Heat Resistance of Spores of *Bacillus amyloliquefaciens* and *Bacillus licheniformis* Results from the Presence of a *spoVA* Operon in a Tn*1546* Transposon

**DOI:** 10.3389/fmicb.2016.01912

**Published:** 2016-12-02

**Authors:** Erwin M. Berendsen, Rosella A. Koning, Jos Boekhorst, Anne de Jong, Oscar P. Kuipers, Marjon H. J. Wells-Bennik

**Affiliations:** ^1^Top Institute Food and NutritionWageningen, Netherlands; ^2^Laboratory of Molecular Genetics, University of GroningenGroningen, Netherlands; ^3^NIZO Food ResearchEde, Netherlands

**Keywords:** spores, heat resistance, Tn*1546* transposon, *spoVA* operon, genome analysis

## Abstract

Bacterial endospore formers can produce spores that are resistant to many food processing conditions, including heat. Some spores may survive heating processes aimed at production of commercially sterile foods. Recently, it was shown that a *spoVA* operon, designated *spoVA*^2mob^, present on a Tn*1546* transposon in *Bacillus subtilis*, leads to profoundly increased wet heat resistance of *B. subtilis* spores. Such Tn*1546* transposon elements including the *spoVA*^2mob^ operon were also found in several strains of *Bacillus amyloliquefaciens* and *Bacillus licheniformis*, and these strains were shown to produce spores with significantly higher resistances to wet heat than their counterparts lacking this transposon. In this study, the locations and compositions of Tn*1546* transposons encompassing the *spoVA*^2mob^ operons in *B. amyloliquefaciens* and *B. licheniformis* were analyzed. Introduction of these *spoVA*^2mob^ operons into *B. subtilis* 168 (producing spores that are not highly heat resistant) rendered mutant 168 strains that produced high-level heat resistant spores, demonstrating that these elements in *B. amyloliquefaciens* and *B. licheniformis* are responsible for high level heat resistance of spores. Assessment of growth of the nine strains of each species between 5.2°C and 57.7°C showed some differences between strains, especially at lower temperatures, but all strains were able to grow at 57.7°C. Strains of *B. amyloliquefaciens* and *B. licheniformis* that contain the Tn*1546* elements (and produce high-level heat resistant spores) grew at temperatures similar to those of their Tn*1546*-negative counterparts that produce low-level heat resistant spores. The findings presented in this study allow for detection of *B. amyloliquefaciens* and *B. licheniformis* strains that produce highly heat resistant spores in the food chain.

## Introduction

The ubiquitous presence of bacterial spore formers in nature can be largely attributed to their ability to produce endospores (spores) that can survive harsh environmental conditions (Nicholson et al., 2000; Setlow, 2006). Bacterial spores can enter the food chain from many different sources, for example via soil, dust, and biofilms (Heyndrickx, 2011). The intrinsic resistance properties of spores may result in survival during food processing, in which heating is one of the most commonly applied treatments to reduce bacterial loads. Such treatments put selective pressure on the microflora that is present, allowing for survival of those strains that produce spores with high heat resistance (Postollec et al., 2012). Surviving spores may germinate upon exposure to certain environmental triggers, and can subsequently resume vegetative growth, potentially resulting in food pathogenicity or food spoilage, depending on the species (Scheldeman et al., 2006; Wells-Bennik et al., 2016).

Spores of mesophilic species belonging to the *B. subtilis* group are commonly found in various food ingredients and food products. The *B. subtilis* group encompasses the species *B. subtilis, B. amyloliquefaciens, B. licheniformis, B. vallismortis, B. mojavensis, B. atropheus*, and *B. sonorensis*, which are phylogenetically close, yet distinguishable (Logan and Vos, 2009). These species can generally grow between temperatures of 30–50°C, with reported growth temperatures of *B. licheniformis* up to 58°C (Warth, 1978). The spores of *B. subtilis, B. amyloliquefaciens* and *B. licheniformis* are commonly found in various food ingredients and food products including cocoa, herbs, spices, bread, soups, milk, and milk powders (te Giffel et al., 1996; Oomes et al., 2007; Lima et al., 2011; Lücking et al., 2013; Miller et al., 2015). These species are for instance well-known contaminants of raw materials used in bread making (Rosenkvist and Hansen, 1995; Sorokulova et al., 2003), and the spores can potentially even survive the bread baking process (Valerio et al., 2015). After spore survival, germination, and outgrowth, vegetative cells of *B. amyloliquefaciens, B. subtilis* or *B. licheniformis* can result in spoiled food products. *B. subtilis*, for instance has been reported to be present in cocoa (Lima et al., 2011) leading to spoiled chocolate drinks, *B. licheniformis* may be present in milk and milk powders leading to spoilage of heat treated dairy products (Gopal et al., 2015), and *B. amyloliquefaciens* may spoil bread, resulting in ropy bread by degradation of starch and the formation of extracellular polysaccharides (Sorokulova et al., 2003; Valerio et al., 2012, 2015). Certain strains of *B. licheniformis* can produce a toxin, lichenisyn A, that can cause foodborne illness (Salkinoja-Salonen et al., 1999; Nieminen et al., 2007; Logan, 2012). Lichenisyn is a non-ribosomally synthesized lipo-peptide that is heat-stable (Konz et al., 1999). Due to the pathogenic potential of strains of *B. licheniformis*, it is critical to control these spores in the food chain (Madslien et al., 2013).

Notable differences have been observed with respect to the spore wet heat resistance properties of strains within the *B. subtilis* group (Kort et al., 2005; Oomes et al., 2007; Lima et al., 2011; Berendsen et al., 2015). Following a detailed analysis of the heat resistance of spores of 14 strains belonging to the *B. subtilis* group, strains could be divided in two groups based on spore heat resistance (Berendsen et al., 2015). For *B. subtilis* strains, it was recently demonstrated that spores with high-level heat resistance contain a Tn*1546* transposon, encompassing a *spoVA* operon that is directly responsible for this phenotype (designated *spoVA*^2mob^, where mob indicates the presence on a mobile genetic element; Berendsen et al., 2016a). In addition, we observed high-level heat resistance of spores of *B. licheniformis* and *B. amyloliquefaciens* strains that carried the Tn*1546* transposon; the spores of these strains showed heat resistance levels similar to as those of spores of *B. subtilis* strains with a Tn*1546* transposon (Berendsen et al., 2015, 2016a).

In this study, we report the presence and composition of Tn*1546* transposon homologs of *B. subtilis* which were found in strains of *B. amyloliquefaciens* and strains of *B. licheniformis* that produced highly heat resistant spores. This was performed by genome analysis or PCR detection. The *spoVA*^2mob^ operons found in *B. amyloliquefaciens* and *B. licheniformis* were introduced into *B. subtilis* to assess their role in spore heat resistance. In addition, the growth temperatures of all *B. amyloliquefaciens* and *B. licheniformis* strains (nine each) with or without the Tn*1546* transposons were assessed.

## Materials and methods

### Bacterial strains used in this study

The strains used in this study for genomic and phenotypic analyses are listed in Table 1. This included nine strains of *B. amyloliquefaciens* isolated from natural and food environments, nine strains of *B. licheniformis* from food environments, and two strains of *B. subtilis*. The genome sequences were available for the two *B. subtilis* strains, all *B. amyloliquefaciens* strains, and four *B. licheniformis* strains (Berendsen et al., 2016a,b; Krawczyk et al., 2016). For *B. amyloliquefaciens* strains B425 and B4140, the heat inactivation kinetics of spores were described previously (Berendsen et al., 2015) and for all other *B. amyloliquefaciens* and *B. licheniformis* strains, the heat inactivation kinetics of spores were previously determined (Berendsen et al., 2016a). Strains of *B. subtilis* 168 and *B. subtilis* B4146 were included in the genome analysis as reference strains, and the heat resistances of the spores of these strains were previously analyzed (Berendsen et al., 2015).

**Table 1:** **Strains used in this study**.

**Strain no**	**Species**	**Description**	**Tn*1546***	**Genome sequence**	**References**
B425	*B. amyloliquefaciens*	Isolated from sterilized milk	+	LQYP00000000	Berendsen et al., 2015; Krawczyk et al., 2016
B4140	*B. amyloliquefaciens*	Isolated from pizza	−	LQYO00000000	Berendsen et al., 2015; Krawczyk et al., 2016
10A5	*B. amyloliquefaciens*	Known as 10A5; NRRL B-14393, isolated from soil	−(PCR)	No	Priest et al., 1988
FZB42	*B. amyloliquefaciens*	Known as 10A6; FZB42, isolated from plant soil	−	NC_009725	Chen et al., 2007
10A18	*B. amyloliquefaciens*	Known as 10A18; CU8004	−(PCR)	No	Zahler et al., 1987
DSM7	*B. amyloliquefaciens*	Known as DSM7, isolated from soil	+	FN597644	Ruckert et al., 2011
DSM1060	*B. amyloliquefaciens*	Known as DSM1060	−(PCR)	No	Priest et al., 1987
101	*B. amyloliquefaciens*	Received as 101	−(PCR)	No	Berendsen et al., 2016a
SB42	*B. amyloliquefaciens*	Received as SB42	−(PCR)	No	Berendsen et al., 2016a
B4089	*B. licheniformis*	Known as E5/T12, isolated from pea soup	−	LKPM00000000	Oomes et al., 2007
B4090	*B. licheniformis*	Known as T1, isolated from pea soup	+	LQYL00000000	Oomes et al., 2007; Krawczyk et al., 2016
B4091	*B. licheniformis*	Known as T29, isolated from mushroom soup	−	LQYM00000000	Oomes et al., 2007; Krawczyk et al., 2016
B4092	*B. licheniformis*	Isolated from buttermilk powder	+	LQYK00000000	Krawczyk et al., 2016
B4094	*B. licheniformis*	Isolated from camomile tea	+	LKPN00000000	Berendsen et al., 2016a
B4121	*B. licheniformis*	Isolated from sateh pastry	−	LKPO00000000	Berendsen et al., 2016a
B4123	*B. licheniformis*	Isolated from sateh pastry	−	LKPP00000000	Berendsen et al., 2016a
B4124	*B. licheniformis*	Isolated from pancakes	−	LKPQ00000000	Berendsen et al., 2016a
B4125	*B. licheniformis*	Isolated from pancakes	−	LKPR00000000	Berendsen et al., 2016a
B4062	*B. subtilis*	Type strain 168	−	NC_000964	Kunst et al., 1997
B4146	*B. subtilis*	Isolated from curry sauce	+	NZ_JXHR01000000	Berendsen et al., 2015, 2016a,b
168-*spoVA*^2mob^ (B4090)	*B. subtilis*	168 *amyE*::*spoVA*^2mob^ (B4090) specR	−	−	This study
168-*spoVA*^2mob^(DSM7)	*B. subtilis*	168 *amyE*::*spoVA*^2mob^ (DSM7) specR	−	−	This study

### Genome analysis

Multiple sequence alignments were made for protein sequences of conserved genes that were present in single copy in all genomes using MUSCLE (Edgar, 2004). The core genome phylogenetic tree was constructed using PHYML (Guindon and Gascuel, 2003). To investigate the presence of the Tn*1546* transposon and the encoded *spoVA* (designated *spoVA*^2mob^) operon that mediates high-level heat resistance, an orthology matrix was constructed using Ortho-MCL (Li et al., 2003) with the genomes of the four *B. amyloliquefaciens* strains, the nine *B. licheniformis* strains, *B. subtilis* B4146 as a strain that produces spores with high heat resistance, and *B. subtilis* 168 as a reference strain that produces spores with low-level heat resistance (Supplementary Data Sheet 1). The genomic organization of the Tn*1546* transposon was visualized using the Artemis comparison tool (ACT), and using microbial genomic context viewer (MGcV) (Carver et al., 2005, 2012; Overmars et al., 2013). For the identified Tn*1546* transposons, operon predictions were performed using FGENESB (http://www.softberry.com). Additionally, manual sequence comparisons and searches for pseudogenes were performed for all genes in the transposon.

Generic PCR primers were used, as described previously, for the detection of the Tn*1546* encoded genes *tnpA, spoVAC*^2mob^ and *cls* in the five strains of *B. amyloliquefaciens* for which no genome sequence information was available (i.e., strains 10A5, FZB42, 10A18, 101 and SB42; Berendsen et al., 2016a).

### Comparison of heat inactivation kinetics of spores

For all strains the heat resistances of their spores (produced at 37°C) was previously determined using capillary tubes, at three different temperatures, using at least five time points, as described in Berendsen et al. (2015) and Berendsen et al. (2016a). For comparison of strain specific heat resistance of spores, the *z*-values (i.e., the increase in temperature required to achieve an additional log unit reduction) were calculated as described previously (van Asselt and Zwietering, 2006). Furthermore, the *D*-values (i.e., the time needed at that temperature to decrease the spore count 10-fold) reported previously, were used to calculate reference *D*-values (*D*_ref_) at the reference temperature of 110°C with the corresponding 95% prediction intervals, as presented in Supplementary Table 1 (van Asselt and Zwietering, 2006).

### Cloning of the *spoVA*^2mob^ operon

The *spoVA*^2mob^ operon, including the predicted promotor region as present in strain *B. licheniformis* B4090 and *B. amyloliquefaciens* DSM7 was cloned into plasmid pDG1730, using a procedure as previously described (Berendsen et al., 2016a). The obtained constructs were transformed to *B. subtilis* 168 and integrated in the *amyE* locus as described in Berendsen et al. (2016a), to verify the role of this operon in increased heat resistance of spores. Spores were prepared for strains 168 *amyE*::*spoVA*^2mob^ (DSM7), 168 *amyE*::*spoVA*^2mob^ (B4090) and 168 as described above, and the heat resistance of spores of these strains was assessed by heating at 100°C for 1 h, followed by plating, incubation and enumeration of survivors.

### Determination of the growth temperature

All strains were cultured from the −80°C glycerol stocks in brain heart infusion broth (BHI, Oxoid). For assessing growth at different temperatures, 24-well plates were filled with 1.5 ml of BHI agar and surface inoculated with 20 μl of turbid individual overnight cultures (>10^7^ cfu mL^−1^, i.e., > 2 × 10^5^ cells on the surface of the agar in each well). Growth or no growth on the agar surface was assessed on day 2, 6, 13, 23, and 30 at the following temperatures: 5.2, 11.5, 14.5, 30.5, 36.3, 45.9, 55.1, and 57.7°C. Growth was scored visually, based on the formation of colonies or a lawn on the agar surface in the wells. The day on which growth was first observed was noted. Two biological replicates were performed, and for each biological replicate, two technical replicates were performed.

## Results

### Genome mining for the Tn*1546* transposon

**Figure 1:**
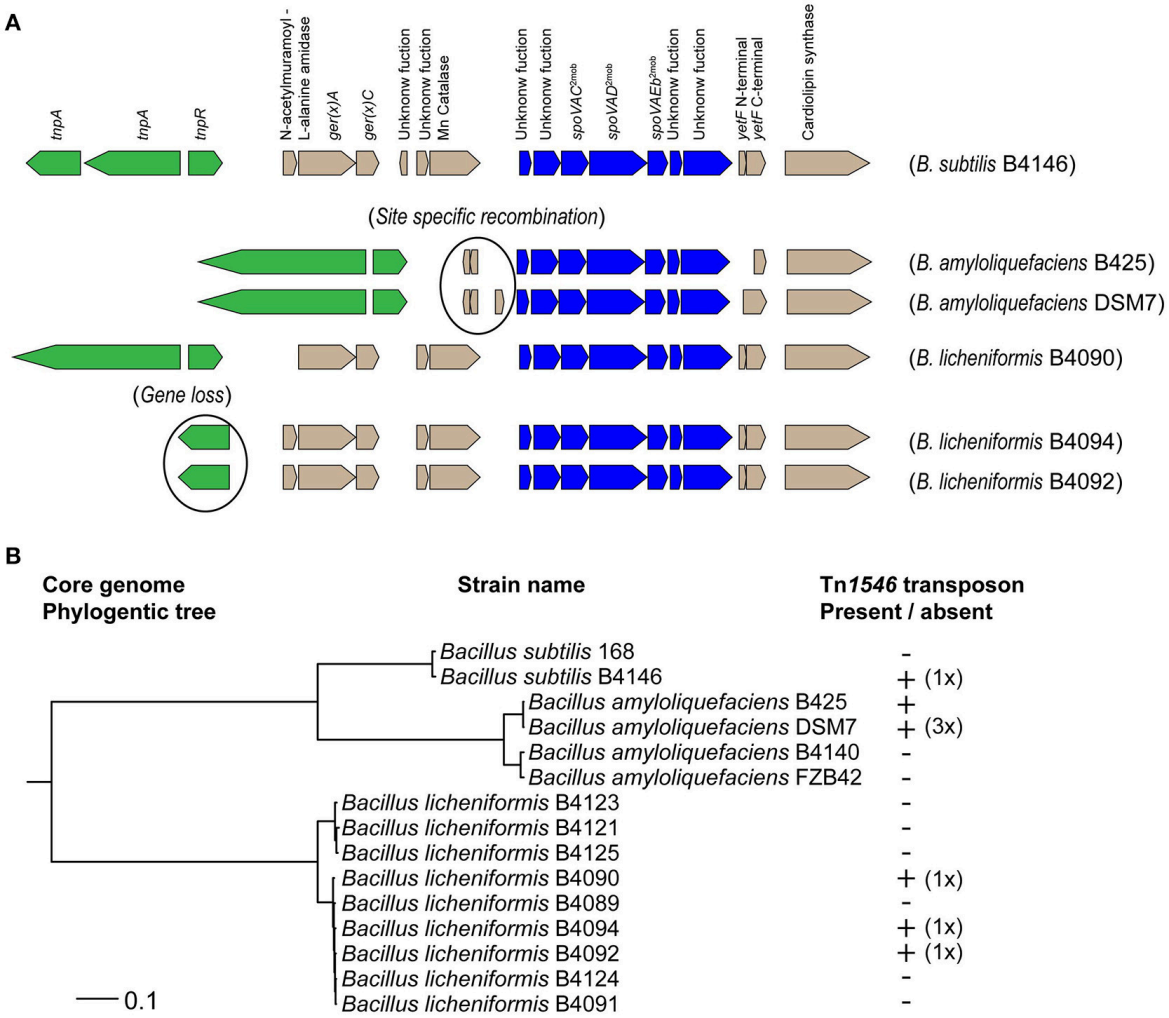
**(A)** Overview of the Tn*1546* transposons found in *B. subtilis* B4146, *B. amyloliquefaciens* B425 and DSM7, and *B. licheniformis* B4090, B4092, and B4090. The predicted gene functions are indicated for the transposon of *B. subtilis* B4146: a transposase gene (*tnpA*), a resolvase gene (*res*), an operon of N-acetylmuramoyl-L-alanine amidase, *gerKA* and *ger*(X)*C* (Operon 1), an operon of a gene with unknown function and a manganese catalase (Operon2), an operon of two genes with unknown functions, *spoVAC, spoVAD, spoVAEb* and two genes with unknown functions (Operon 3, *spoVA*^2mob^), a fragmented *yetF* gene (Gene 4), and a cardiolipin synthase gene (Gene 5). The transposons in *B. amyloliquefaciens* are smaller, probably due to site specific recombination events, whereby operon 1 and operon 2 were lost. In *B. licheniformis* B4092 and B4094 the resolvase gene was not present, which is most likely a result of gene loss. **(B)** Maximum likelihood phylogenetic tree based on core genome of single genes of 15 strains of the *B. subtilis* group. The presence and copy number of the Tn*1546* transposon was indicated behind the corresponding strains. *B. subtilis* B4146, *B. licheniformis* B4090, B4092, and B4094 carry a single transposon. The *B. amyloliquefaciens* strain DSM7 carries three copies of the transposon, while for strain B425 the copy number could not be determined.

For *B. subtilis* strains, it has been demonstrated that the presence of a Tn*1546* transposon is responsible for high-level heat resistance of the spores (Berendsen et al., 2016a). The presence of the Tn*1546* transposon was assessed in nine strains of *B. amyloliquefaciens* and nine strains of *B. licheniformis*. The genome sequences of all nine strains of *B. licheniformis* were available and in three of these strains the Tn*1546* transposon was found (namely, strains B4090, B4092, and B4094; Figure 1). The genome sequences of four strains of *B. amyloliquefaciens* were available, and the Tn*1546* transposon was found in two of these strains of *B. amyloliquefaciens* (namely in B425 and DSM7; Figure 1). The predicted protein for the transposase TnpA, which is part of the Tn*1546* transposon, was found in orthologous group OG3133 for all of the strains that carry the Tn*1546* transposon. Genome sequences were not available for the other five strains of *B. amyloliquefaciens*. PCR-based detection of the genes *tnpA, spoVAC* and *cls* (that are present on the transposon and very well conserved) using primers for these three target genes showed positive results for *B. amyloliquefaciens* strains B425 and DSM7 but did not reveal the transposon in the other five strains. In short, two out of nine strains of *B. amyloliquefaciens* and three out of nine strains of *B. licheniformis* contained the Tn*1546* transposon.

### Heat resistance of spores is related to the presence of the Tn*1546* transposon

The heat resistances of spores of *B. amyloliquefaciens* and *B. licheniformis* were assessed in relation to the presence or absence of the Tn*1546* transposon (Supplementary Table 1). The heat resistance of spores was displayed as a reference decimal reduction time of spores per strain at the reference temperature of 110°C ($D_{110^{{}^{\circ}}C}$-values) in relation to the presence or absence of the Tn*1546* transposon (Supplementary Table 1).

Two strains of *B. amyloliquefaciens*, namely B425 and DSM7, contained the Tn*1546* transposon. These strains produced spores that required significantly longer heating times (unpaired *t*-test *p* < 0.001) at 110°C, i.e., approximately 15 times, to achieve one decimal reduction, than spores of the other seven strains without this transposon. Strains *B. licheniformis* B4090, B4092, and B4094 contained the Tn*1546* transposon, and the spores of these strains all required longer heating times (unpaired *t*-test *p* < 0.001) (2.5 times) to reach a decimal reduction than the spores of the six *B. licheniformis* strains that did not possess the Tn*1546* transposon.

### The *spoVA*^2mob^ operon is responsible for increased heat resistance of spores

**Figure 2:**
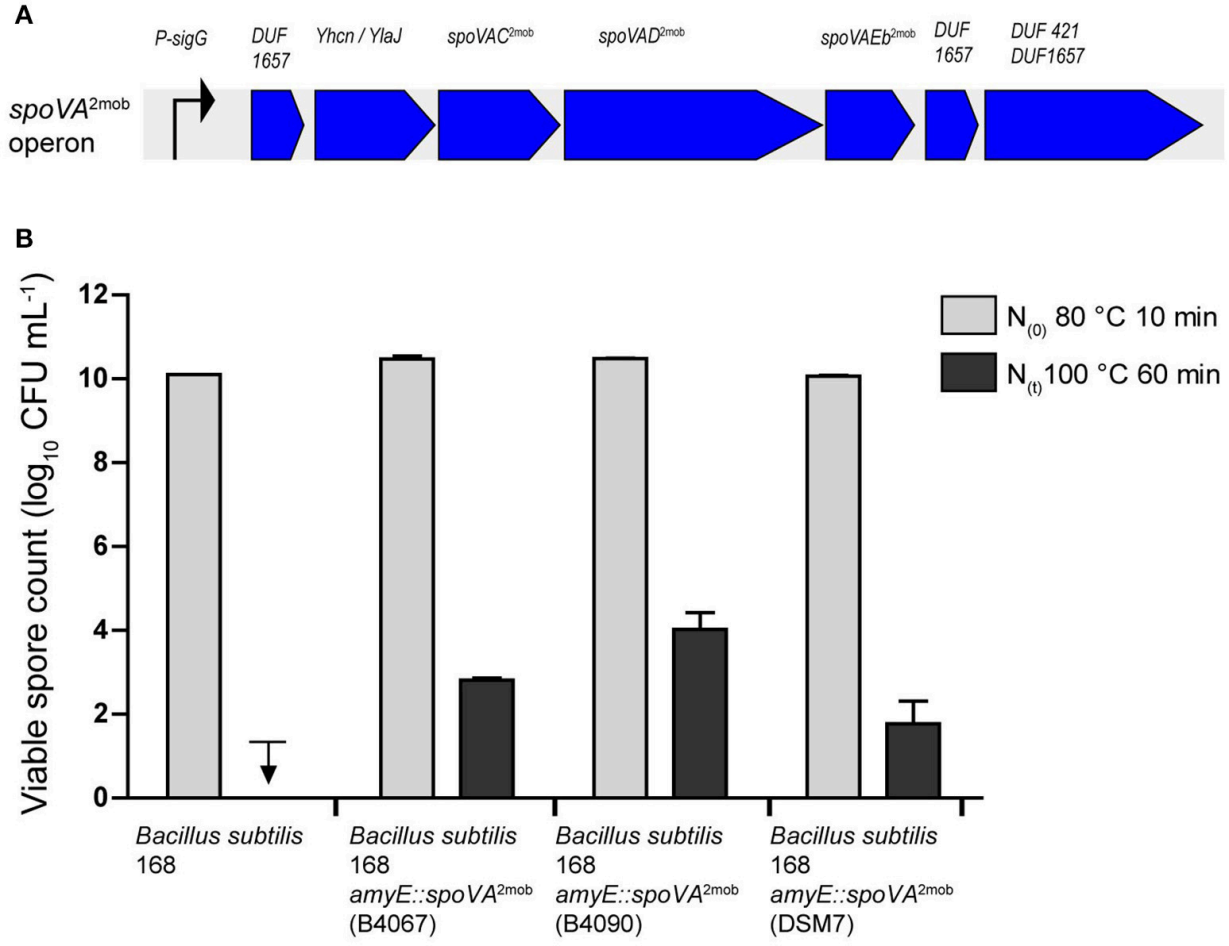
**(A)** Overview of the *spoVA*^2mob^ operon, as initially found in *B. subtilis* strain B4146. The *spoVA*^2mob^ operon has a predicted sigma G binding site upstream of the first gene. The operon consists of seven genes: a gene of unknown function with a predicted DUF1657 domain, a gene of unknown function with a predicted YhcN/YlaJ domain, *spoVAC, spoVAD, spoVAEb*, a gene of unknown function with a predicted DUF1657 domain, and a gene of unknown function with a predicted DUF421 and a DUF1657 domain. **(B)** Spore counts after heating at 80°C for 10 min and at 100°C for 60 min for strains of *B. subtilis* 168, *B. subtilis* 168 *amyE*::*spoVA*^2mob^ [B4067, data from Berendsen et al. (2016a)], *B. subtilis* 168 *amyE*::*spoVA*^2mob^ (B4090), and *B. subtilis* 168 *amyE*::*spoVA*^2mob^ (DSM7). The downward arrow indicates that the spores were inactivated below detection limit.

We previously showed that the introduction of the *spoVA*^2mob^ operon originating from *B. subtilis* strain B4067 into laboratory strain *B. subtilis* 168 resulted in the formation of high-level heat resistant spores by this strain (Berendsen et al., 2016a). To establish whether the *spoVA*^2mob^ operons present in the Tn*1546* transposons of *B. licheniformis* B4090 and *B. amyloliquefaciens* DSM7 have a functional role, these operons were also introduced individually into *B. subtilis* 168. The *B. subtilis* 168 mutants carrying the *spoVA*^2mob^ genes of *B. licheniformis* B4090 and the *spoVA*^2mob^ genes of *B. amyloliquefaciens* produced spores with significantly higher heat resistances than the parent strains after heating at 100°C for 60 min (Figure 2). The spores of *B. subtilis* 168 were inactivated to a level below the detection limit, i.e., more than 8 log units reduction, indicative of a low level of heat resistance of spores. The spores of *B. subtilis* 168 *amyE*::*spoVA*^2mob^ (containing the *spoVA*^2mob^ operon of *B. licheniformis* B4090) and spores of *B. subtilis* 168 *amyE*::*spoVA*^2mob^ (containing the *spoVA*^2mob^ operon of *B. amyloliquefaciens* DSM7) showed survival of 4.0 log_10_ unit (±0.4), and 1.7 log_10_ unit (±0.5), respectively, indicating a high level of heat resistance of spores. The control strain *B. subtilis* 168 *amyE*::*spoVA*^2mob^ producing high level heat resistant spores (containing the *spoVA*^2mob^ operon of *B. subtilis* B4067) (Berendsen et al., 2016a), showed survival of 2.8 log_10_ unit (±0.05).

### Detailed analysis of the Tn*1546* transposon

The composition of the Tn*1546* transposon in *B. licheniformis* strains B4090, B4092 and B4094 is shown in Figure 1A. In these strains, the Tn*1546* transposon is highly similar to the one found in *B. subtilis* B4146 (Figure 1A). The transposons found in *B. subtilis* and *B. licheniformis* consist of genes that are required for transposition, and furthermore contain three operons and two single genes. The Tn*1546* transposon found in the *B. amyloliquefaciens* strains DSM7 and B425 was smaller than the transposon found in *B. subtilis* and *B. licheniformis.* In both *B. amyloliquefaciens* strains, the first two operons were absent, possibly due to a site-specific recombination event, as a recombinase gene and a hypothetical gene were present at that genomic location.

The evolutionary relatedness of the different strains and species was visualized in a maximum likelihood core genome phylogenetic tree, based on concatenated protein sequences of conserved genes present in single copy in all genomes (Figure 1B). The species *B. amyloliquefaciens, B. licheniformis* and *B. subtilis* clustered in separate branches of the phylogenetic tree. For *B. amyloliquefaciens*, the strains with the Tn*1546* transposon clustered together, whereas this was not the case for *B. licheniformis* strains carrying the Tn*1546* transposon.

The genomic locations of the Tn*1546* transposons were different for *B. subtilis, B. amyloliquefaciens* and *B. licheniformis*. In *B. subtilis*, the transposon was found at two genomic locations, namely inserted in *yitF* and between *yxjA* and *yxjB* (Berendsen et al., 2016a). In *B. amyloliquefaciens*, three Tn*1546* transposons were found in strain DSM7 at three different genomic locations, namely between a gene encoding for a fructose-1,6-bisphophatase and a hypothetical gene, between two hypothetical genes, and between a hypothetical gene and *rapK*. For *B. amyloliquefaciens* strain B425, it was not possible to determine the genomic location(s) and copy number of the Tn*1546* transposon, since contig breaks were present on both sides of the Tn*1546* transposon. In *B. licheniformis* strains B4090, B4092, and B4094, a single Tn*1546* transposon was found integrated in a gene that encodes a D-alanyl-D-alanine carboxypeptidase.

Detailed analysis revealed that some genes in the Tn*1546* transposon were mutated and present as pseudogenes in the transposon of some strains. The genes *tnpA* and *tnpR* in the Tn*1546* transposon (which are required for active transposition) were intact and present in *B. amyloliquefaciens* strains B425 and DSM7 and in *B. licheniformis* strain B4090.

### Determination of vegetative growth at different temperatures

None of the 18 *B. amyloliquefaciens* and *B. licheniformis* strains were able to grow at 5.2°C within 30 days. At 11.5°C, six out of nine *B. amyloliquefaciens* strain and five out of nine *B. licheniformis* strains showed growth. At 14.5°C, all but one strain showed growth. All strains grew at the highest temperature tested (57.7°C), and at all other temperatures, growth of strains was observed except for strain 101 at 55.1°C. In the case of the nine *B. amyloliquefaciens* and nine *B. licheniformis* strains, high level heat resistance of spores due to the presence of the Tn*1546* transposon was not correlated with the ability to grow at different temperatures (Table 2).

**Table 2:** **Determination of ability to grow at different temperatures for nine strains of *B. amyloliquefaciens* and nine strains of *B. licheniformis***.

**Species**	**Strain**	**Tn*1546* transposon**	**Growth observed at temperature^a^**							
			**5.2°C**	**11.5°C**	**14.5°C**	**30.5°C**	**36.3°C**	**45.9°C**	**55.1°C**	**57.7°C**
*B. amyloliquefaciens*	10A5	No	−	+++	+++	++++	++++	++++	+++	+++
	FZB42	No	−	−	−	++++	++++	++++	++++	+++
	10A18	No	−	−	+++	++++	++++	++++	++++	++++
	101	No	−	+++	+++	++++	++++	++++	−	+++
	SB42	No	−	++	++++	++++	++++	++++	+++	+++
	B4140	No	−	+++	++++	++++	++++	++++	++	++++
	DSM1060	No	−	−	++	++++	++++	++++	+++	++++
	DSM7	Yes	−	++	+++	++++	++++	++++	++++	++++
	B425	Yes	−	+++	+++	++++	++++	++++	++	++++
*B. licheniformis*	B4089	No	−	−	++	++++	++++	++++	++++	+++
	B4091	No	−	−	+++	++++	++++	++++	++++	++++
	B4121	No	−	−	+++	++++	++++	++++	+++	+++
	B4123	No	−	+++	+++	++++	++++	++++	++++	+++
	B4124	No	−	++	+++	++++	++++	++++	++++	+++
	B4125	No	−	+	+++	++++	++++	++++	+++	+++
	B4090	Yes	−	+	++	++++	++++	++++	++++	+++
	B4092	Yes	−	+++	+++	++++	++++	++++	++++	++++
	B4094	Yes	−	−	++	++++	++++	++++	++++	+++

a *Growth in: 2 days (++++), 6 days (+++), 13 days (++), 23 days (+), and no growth within 30 days(−)*.

## Discussion

*B. licheniformis* strains B4090, B4092, and B4094 contained a single copy of the Tn*1546* transposon with a single *spoVA*^2mob^ operon. The heat resistances of spores of *B. licheniformis* with or without this operon were significantly different, but relatively modest. For *B. amyloliquefaciens*, spores of strains B425 and DSM7 showed comparable high-levels of heat resistance, which were significantly higher than those of the spores of other *B. amyloliquefaciens* strains. Strain DSM7 contains three Tn*1546* transposable elements, and it is likely that strain B425 also contains multiple copies, however this remains to be established. The number of *spoVA*^2mob^ operons have previously been found to correlate with the level of heat resistance of spores in *B. subtilis*; strains carrying three copies produced spores with the highest level of heat resistance (Berendsen et al., 2016a). For *B. subtilis*, it has been shown that the Tn*1546* transposon was found at different locations in the genome, all leading to high-level heat resistance of spores (Berendsen et al., 2016a). It remains to be established whether the location of the insertion of the transposon in the genome of *B. amyloliquefaciens* and *B. licheniformis* plays a role in the level of heat resistance of spores.

The presence of intact *tnpA* and *tnpR* genes in *B. amyloliquefaciens* DSM7 and B425, and *B. licheniformis* B4090 suggests that active transposition of the Tn*1546* element may be possible for these strains, although active transposition of the Tn*1546* transposon is believed to require a plasmid intermediate, as has been described in *Enterococcus faecium* (Arthur et al., 1993). Interestingly, *B. amyloliquefaciens* DSM7, containing the intact *tnpA* and *tnpR* genes, contained three Tn*1546* transposons. The encoded proteins required for transposition potentially allowed for internal transposition within the chromosome of strain DSM7. In *B. subtilis* strain B4146 and in *B. licheniformis* strains B4092 and B4094, the transposition genes are absent or not intact, suggesting that active transposition of the Tn*1546* transposon is not likely to occur in these strains. This does not mean that the transposons cannot be transferred; transfer of genetic material including the Tn*1546* transposon can be mediated by other transfer mechanisms, such as phage transduction, as described previously for *B. subtilis* (Berendsen et al., 2016a), or via the uptake of external DNA via natural competence (Kovacs et al., 2009).

The *spoVA*^2mob^ operons present on the Tn*1546* transposons differ from the *spoVA* operons (designated *spoVA*^1^) that are encoded on the chromosomes of *B. subtilis* (Tovar-Rojo et al., 2002), *B. amyloliquefaciens* and *B. licheniformis*. The SpoVA proteins encoded in the *spoVA*^1^ operon are required for uptake of DPA during the sporulation process, and sporulation cannot be completed upon deletion or disruption of these genes in *B. subtilis* 168 (Tovar-Rojo et al., 2002). In addition, the SpoVA proteins are involved in the release of Ca-DPA during the germination process (Vepachedu and Setlow, 2004, 2007), with the SpoVAC protein functioning as a mechano sensitive channel during germination (Velasquez et al., 2014). The SpoVAD protein has a binding pocket, whereby it can directly bind DPA (Li et al., 2012). Both the *spoVA*^1^ and the *spoVA*^2mob^ operons contain genes encoding SpoVAC, SpoVAD and SpoVAEb, while the other genes in the operons are different. Presumably the presence of the *spoVA*^2mob^ encoded proteins results in the increased uptake of DPA into the spore core, as was previously found for *B. subtilis* (Berendsen et al., 2016a).

It is known that environmental conditions during sporulation, such as temperature, matrix and medium composition, can influence the heat resistance of spores of the *B. subtilis* group (Cazemier et al., 2001; Melly et al., 2002; Rose et al., 2007). To allow for a direct comparison of the resistance properties of spores of the different strains, the applied sporulation conditions of all *B. licheniformis* and *B. amyloliquefaciens* strains were the same (Berendsen et al., 2015, 2016a). Factors that are known to influence spore heat resistance include the composition of the sporulation medium, and it is known that the addition of salts (Ca^2+^, Mn^2+^, Mg^2+^ and K^+^) results in higher resistances of spores to heat (Cazemier et al., 2001). Furthermore, the temperature during sporulation can influence the heat resistance of *B. subtilis* spores (Melly et al., 2002). In line with these findings, the heat resistances of spores of a *B. licheniformis* strain have been reported to be higher upon sporulation at 45°C, with a modeled optimum at 49°C, than at lower temperatures such as 20°C (Baril et al., 2012). Overall, the environmental conditions during sporulation, the presence or absence of genetic elements such as the *spoVA*^2mob^ operon, and the storage conditions of spores will ultimately determine the heat resistance properties of spores. It is therefore conceivable that spores produced under laboratory conditions do not necessarily reach the same levels of heat resistance as spores found in foods (van Zuijlen et al., 2010; Lima et al., 2011).

It is generally assumed for bacterial spore formers that higher optimal growth temperatures of vegetative cells correlates positively with spore heat resistance (Nicholson et al., 2000). In this study, some differences were seen between strains with respect to their abilities to grow at different temperatures, but no consistent pattern was seen for strains that contain the Tn*1546* element and produce high level heat resistant spores versus the ones that do not harbor the element and produce low-level heat resistant spores. All strains were able to grow at 57.7°C. All genes present on the Tn*1546* transposon are under the control of sporulation-specific sigma factors K or G (Berendsen et al., 2016a) and are thus only expressed during late stages of sporulation. It is therefore in line with expectations that the genes on the Tn*1546* transposon, which determine spore heat resistance, do not influence the ability of vegetative cells to grow at high temperatures as these genes are not expressed during vegetative growth.

## Conclusions

Variation in heat resistance of spores exists between strains of different spore forming species but also within species (Oomes et al., 2007; Orsburn et al., 2008; Lima et al., 2011; Berendsen et al., 2015). In this study, a genomic analysis revealed the presence of Tn*1546* transposons in two strains of *B. amyloliquefaciens* and in three strains of *B. licheniformis*. The presence of this transposon, containing the *spoVA*^2mob^ operon, correlated with high-level heat resistance of spores. Strains producing low or high level heat resistant spores showed similar temperature ranges for growth. A functional role of the *spoVA*^2mob^ operon in increasing the heat resistance of spores was demonstrated by cloning these operons in *B. subtilis* 168, resulting in spores with high-level heat resistance. Clearly, mere identification of the species of spores in food products does not provide information on the heat resistance levels of these spores. The knowledge obtained in this study on the role of the *spoVA*^2mob^ operon in spore heat resistance can be used for specific detection of strains of the *B. subtilis* group that produce high-level heat resistant spores. Multiple DNA based methods can be used for the detection of such genetic elements, such as whole genome sequencing and specific PCR detection, among others (Caspers et al., 2011; Alkema et al., 2016). The ability to detect certain strains of *B. subtilis* group that have the ability to produce high level heat resistant spores can aid control of these sporeformers in the food chain.

## Author contributions

EB and RK collected the data. EB, RK, AD, and JB analyzed the data. EB, OK, and MW wrote the manuscript.

### Conflict of interest statement

The authors declare that the research was conducted in the absence of any commercial or financial relationships that could be construed as a potential conflict of interest.

## Supplementary material

Supplementary Datasheet 1 **Orthology matrix constructed using Ortho-MCL with the genomes of the four *B. amyloliquefaciens* strains, the nine *B. licheniformis* strains, *B. subtilis* B4146 as a strain that produces spores with high heat resistance and *B. subtilis* 168 as a reference strain that produces spores with normal heat resistance**.

Supplementary Table 1 **Calculated reference *D*-value (*D*_ref_) at a reference temperature (T_ref_) of 110°C, and *z*-value, of spores of nine strains of *B. amyloliquefaciens* and spores of nine strains of *B. licheniformis* (Berendsen et al., 2015, 2016a)**. In these studies, all strains were sporulated on the same media using the same incubation, namely using Nutrient Agar (Difco, 23g/L) plates supplemented with CaCl_2_ (1 mM), KCl (13 mM), MgSO_4_ (1 mM) and MnSO_4_ (0.13 mM), with a pH of 7.0, and incubation for 7 days at 37°C to allow for sporulation (Berendsen et al., 2015). Subsequently spores were harvested from the plates and washed three times with sterile water (5000 *g*, 10 min, 4°C) (Berendsen et al., 2015).
