# Supplementary material for: High-Level Heat Resistance of Spores of Bacillus amyloliquefaciens and Bacillus licheniformis Results from the Presence of a spoVA Operon in a Tn1546 Transposon
Source: Front Microbiol. 2016 Dec 2;7:1912. doi: 10.3389/fmicb.2016.01912 (PMC5133452; doi:10.3389/fmicb.2016.01912)
Supplement: Supplementary Table 1 — Calculated reference D-value (Dref) at a reference temperature (Tref) of 110°C, and z-value, of spores of nine strains of B. amyloliquefaciens and spores of nine strains of B. licheniformis (Berendsen et al., 2015, 2016a). In these studies, all strains were sporulated on the same media using the same incubation, namely using Nutrient Agar (Difco, 23g/L) plates supplemented with CaCl2 (1 mM), KCl (13 mM), MgSO4 (1 mM) and MnSO4 (0.13 mM), with a pH of 7.0, and incubation for 7 days at 37°C to allow for sporulation (Berendsen et al., 2015). Subsequently spores were harvested from the plates and washed three times with sterile water (5000 g, 10 min, 4°C) (Berendsen et al., 2015). [file Table1.docx]

**Supplementary Table 1.** Calculated reference *D*-value (*D*_ref_) at a reference temperature (T_ref_) of 110°C, and z-value, of spores of nine strains of *B. amyloliquefaciens* and spores of nine strains of *B. licheniformis* (Berendsen *et al.*, 2015 and Berendsen *et al.*, 2016a). In these studies, all strains were sporulated on the same media using the same incubation, namely using Nutrient Agar (Difco, 23g/L) plates supplemented with CaCl_2_ (1mM)_,_ KCl (13mM), MgSO_4_ (1mM) and MnSO_4_ (0.13mM), with a pH of 7.0, and incubation for 7 days at 37°C to allow for sporulation (Berendsen *et al.*, 2015). Subsequently spores were harvested from the plates and washed three times with sterile water (5000 *g*, 10 minutes, 4°C) (Berendsen *et al.*, 2015).

| **Species** | **Strain** | **Tn*1546*** | **Inactivation temperatures**  **(°C)** | ***D*_ref 110 °C_ (min)** | **95% upper PI** | **95% lower PI** | ***z*-value (°C)** | **S.E.** |
| --- | --- | --- | --- | --- | --- | --- | --- | --- |
| *B. amyloliquefaciens* | 10A5 | No | 100, 105, 110 | 1.02 | 2.10 | 0.49 | 11.13 | 1.09 |
|  | 10A6 | No | 100, 105, 110 | 0.54 | 1.05 | 0.28 | 13.88 | 1.54 |
|  | 10A18 | No | 100, 105, 110 | 0.61 | 4.29 | 0.09 | 10.32 | 2.53 |
|  | 101 | No | 100, 105, 110 | 0.55 | 0.81 | 0.37 | 11.66 | 0.88 |
|  | SB42 | No | 100, 105, 110 | 0.59 | 0.82 | 0.43 | 11.44 | 0.68 |
|  | B4140^a^ | No | 100, 105, 110 | 0.13 | 0.16 | 0.11 | 7.21 | 0.17 |
|  | DSM1060 | No | 100, 105, 110 | 0.41 | 0.49 | 0.34 | 12.00 | 0.33 |
|  | DSM7 | Yes | 110, 115, 120 | 5.71 | 64.44 | 0.51 | 8.48 | 2.12 |
|  | B425^a^ | Yes | 110, 115, 120 | 10.80 | 21.10 | 5.53 | 6.23 | 0.41 |
| *B. licheniformis* | B4089 | No | 105, 110, 115, 120 | 0.50 | 3.72 | 0.07 | 9.99 | 2.31 |
|  | B4091 | No | 105, 110, 115, 120 | 0.58 | 3.07 | 0.11 | 9.53 | 1.74 |
|  | B4121 | No | 105, 110, 115, 120 | 0.65 | 3.18 | 0.13 | 9.33 | 1.45 |
|  | B4123 | No | 105, 110, 115, 120 | 0.50 | 1.54 | 0.16 | 9.97 | 1.30 |
|  | B4124 | No | 105, 110, 115, 120 | 0.72 | 5.74 | 0.09 | 10.01 | 2.18 |
|  | B4125 | No | 105, 110, 115, 120 | 0.69 | 1.84 | 0.26 | 16.32 | 2.72 |
|  | B4090 | Yes | 110, 115, 120 | 1.48 | 2.16 | 1.02 | 22.10 | 3.01 |
|  | B4092 | Yes | 110, 115, 120 | 1.64 | 2.15 | 1.25 | 16.07 | 0.85 |
|  | B4094 | Yes | 110, 115, 120 | 1.24 | 2.94 | 0.52 | 12.51 | 1.65 |
